# Nebula: ultra-efficient mapping-free structural variant genotyper

**DOI:** 10.1093/nar/gkab025

**Published:** 2021-01-27

**Authors:** Parsoa Khorsand, Fereydoun Hormozdiari

**Affiliations:** Genome Center, UC Davis, Davis, California, 95616, USA; Genome Center, UC Davis, Davis, California, 95616, USA; UC Davis MIND Institute, Sacramento, California, 95817, USA; Department of Biochemistry and Molecular Medicine, UC Davis, Sacramento, California, 95817, USA

## Abstract

Large scale catalogs of common genetic variants (including indels and structural variants) are being created using data from second and third generation whole-genome sequencing technologies. However, the genotyping of these variants in newly sequenced samples is a nontrivial task that requires extensive computational resources. Furthermore, current approaches are mostly limited to only specific types of variants and are generally prone to various errors and ambiguities when genotyping complex events. We are proposing an ultra-efficient approach for genotyping any type of structural variation that is not limited by the shortcomings and complexities of current mapping-based approaches. Our method Nebula utilizes the changes in the count of *k*-mers to predict the genotype of structural variants. We have shown that not only Nebula is an order of magnitude faster than mapping based approaches for genotyping structural variants, but also has comparable accuracy to state-of-the-art approaches. Furthermore, Nebula is a generic framework not limited to any specific type of event. Nebula is publicly available at https://github.com/Parsoa/Nebula.

## INTRODUCTION

Structural variants (SVs) are defined as medium and large size (>50 bp) genomic alterations. SVs have many different types, e.g. deletions, insertions, duplication, transposon insertions and inversions (1–4). It has become clear that SVs are a major contributing factor in diseases (5) and evolution (6). However, efficient and accurate genotyping of all types of SVs using whole-genome sequencing (WGS) data is not a trivial task. In many of the large scale genomic studies SVs are being ignored or are merely an afterthought. One of the main reasons behind SVs not being as thoroughly studied as other types of variants such as SNVs, is due to complexity of efficient and accurate discovery and genotyping of these types of variants. It is hypothesized that lack of comprehensive study of SVs is one of the contributing factors among others to the missing heritability gap observed in complex disorders (7,8).

The advent of high-throughput sequencing (HTS) technologies has made it possible to understand the contribution of SVs in diseases and evolution. In the 1000 Genomes Project (1KG) more than 42 000 SVs were discovered and genotyped in over 2500 samples (9). Recently, a few samples (e.g. CHM1 and CHM13) were sequenced using long-read technologies (i.e. PacBio). A comparison of the SV predictions made using the state-of-the-art computational methods (e.g. LUMPY (10), DELLY (11), TARDIS (12) and Pindel (13)) using the short-read HTS data against the calls produced using long-read data indicated that many SVs ($>50\%$) are missed by our best practices using short-read sequencing data (14). Thus, we are in need of approaches which can efficiently genotype these newly found SVs in a large number of WGS samples.

With WGS data of additional samples being produced at a breathtaking rate, an approach to accurately and efficiently genotype the (common) SVs in newly sequenced samples is needed. In addition, with more comprehensive sets of SVs being predicted using long-read technologies we would like to be able to genotype these newly discovered SVs in the samples that have been already processed.

The current approaches for genotyping SVs using WGS data are mainly based on first mapping the reads to the reference genome and then predicting the genotype (15,16). This framework has three main drawbacks. First, the mapping step is resource intensive. Second, these approaches are mostly tailored to specific types of variants (SNV, small indels and large CNVs). Third, genotyping any variant close to repeats in the reference genome would be less accurate due to the potential of inaccurate mapping.

Mapping-free approaches are becoming popular for different genomic and transcriptome applications. The mapping-free approaches are not limited by the shortcomings of the mapping algorithms and tend to be much more efficient. Mapping-free transcriptome analysis tools such as Kallisto (17) and Salmon (18) have been very helpful in efficient and accurate quantification of RNAseq data. These approaches have also been recently utilized successfully in variant discovery.

One of the first tools to introduce a mapping-free method for variant discovery is Cortex (19). Cortex introduces the concept of colored de bruijn graphs to compare the *k*-mers from different samples to predict variants between the samples (19). Cortex was also used successfully for predicting variants in the 1000 Genomes Project. The method DISCOSNP (20) was one of the first approaches developed for predicting SNPs efficiently using *k*-mers counts. This approach was later developed into DISCOSNP++ to predict indels between multiple sequenced samples using raw unassembled reads (21).

Mapping-free approaches have also have gained traction in predicting somatic variants between a normal sample and the matching tumor. NovoBreak (22) is one such tool that utilizes *k*-mer counts to predict different types of somatic variants between tumor and normal samples using whole-genome sequencing data. Another application of mapping-free approaches is discovery of *de novo* variants in families. The tools Scalpel (23), COBASI (24) and Kevlar (25) are mapping free approach for accurate discovery of *de novo* variants using whole-exome sequenced or whole-genome sequenced samples.

Fast and accurate genotyping of common variants is another recent application of the mapping-free framework. The tools LAVA (26) and VarGeno(27) are developed for fast genotyping of common SNPs using *k*-mer counts. Furthermore, the tool MALVA (28) is a recent mapping-free method for genotyping both SNPs and indels.

Finally, mapping-free approaches have also been utilized in improving the association studies using whole-genome sequencing data (29). The tool HAWK (29) is capable of fast and accurate discovery of variants associated with phenotypes of interest by comparing the *k*-mers frequencies between cases and controls.

The growing list of mapping-free methods and their applications has also resulted in development of several tools for fast and accurate *k*-mer quantification. Some of the tools used for fast *k*-mer quantification include JellyFish (30), Khmer (31), DSK (32) and KMC (33).

Here we are proposing a novel mapping-free approach, Nebula, that utilizes *k*-mer counts for efficient and accurate genotyping of (common) SVs in any whole-genome sequenced sample.

## METHODS

**Figure 1.**
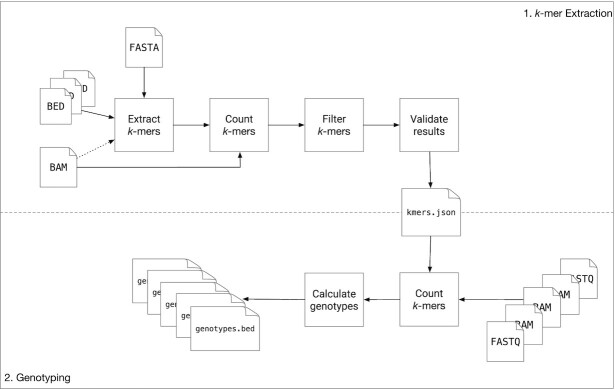
An overview of the entire Nebula pipeline. The upper half shows the *k*-mer extraction stage which takes as input a set of SV coordinates, the reference assembly, and a set of samples on which the genotypes of these SVs is known. The *k*-mer extraction stage selects a collection of *k*-mers to be used for genotyping. The bottom half shows the SV genotyping phase, which uses the *k*-mers extracted earlier to genotype the input SVs on any number of newly sequenced samples without mapping the reads.

Nebula is a mapping-free approach for accurate and efficient genotyping of SVs. Nebula is a two-stage approach and consists of a *k***-**mer extraction phase and a genotyping phase (Figure 1). Given as input a set of SV coordinates (BED/VCF), the reference assembly (FASTA), and a set of mapped samples on which the genotype of the input SVs is already known (BAM), Nebula extracts a collection of *k*-mers that represent the input SVs (*k*-mer extraction phase). These extracted *k*-mers will then be used to genotype the same set of SVs on any new WGS sample(s) without the need to map the reads to the reference genome (genotyping phase). This is done by counting the *k*-mers in the WGS reads of the new sample(s) and predicting genotypes using a likelihood model.

### Likelihood model

The key assumption in Nebula is that each SV will increase and/or decrease the copy number of a specific set of *k*-mers in the genome. Note that the count of each *k*-mer in the WGS reads of a sample is directly correlated with the copy number of the *k*-mer in the corresponding genome. We develop a likelihood model to calculate the probability of different genotypes ({0/0, 0/1, 1/1}) per SV based on the counts of *k*-mers.

We define a unique *k*-mer as one that appears in exactly one loci in the sample’s genome. For a given sample, we assume the number of reads containing a unique *k*-mer that are coming from each haplotype to follow a normal distribution $\mathcal {N}(\mu _h, \sigma ^2_h)$. We also model the total number of reads containing that *k*-mer (i.e., the *k*-mer’s count) in a diploid genome as the summation of the two normal distributions representing the number of reads containing the *k*-mer in each haplotype as $\mathcal {N}(\mu , \sigma ^2) = \mathcal {N}(\mu _1, \sigma _1^2) + \mathcal {N}(\mu _2, \sigma _2^2)$ where $\mu_i$ and $\sigma ^2_i$ are mean and variance for the corresponding haplotype. However as we generally don’t know which haplotype a sequencing read is from, we will directly estimate the sample-wide parameters $\mu\ {\rm and}\ \sigma^2$ rather than the haplotype-specific ones. For this, we select a large number of unique *k*-mers from conserved regions of the genome (e.g. exons) and count them in the sequencing reads of the sample. By further assuming that the sequencing coverage is equal for both haplotypes, the count of a unique *k*-mer present on only one haploid can be approximated using the normal distribution $\mathcal {N}(\mu /2, \sigma ^2/2)$. Finally, the count of a *k*-mer not expected to be present in the genome is estimated by setting $\mu$ to zero and using a small fixed number for the variance. This provides the basis of the model that we use to calculate likelihood of SVs genotypes based on the *k*-mer counts.

### *k*-mer extraction

Nebula uses the coordinates of the input SVs, the reference genome and mapped reads of WGS sample(s) on which the genotype of the SVs of interest are known to extract *k*-mers whose copy number is affected by the SVs. These *k*-mers either cross the SV’s breakpoint or fall inside the region that is affected by the SV.

**Figure 2.**
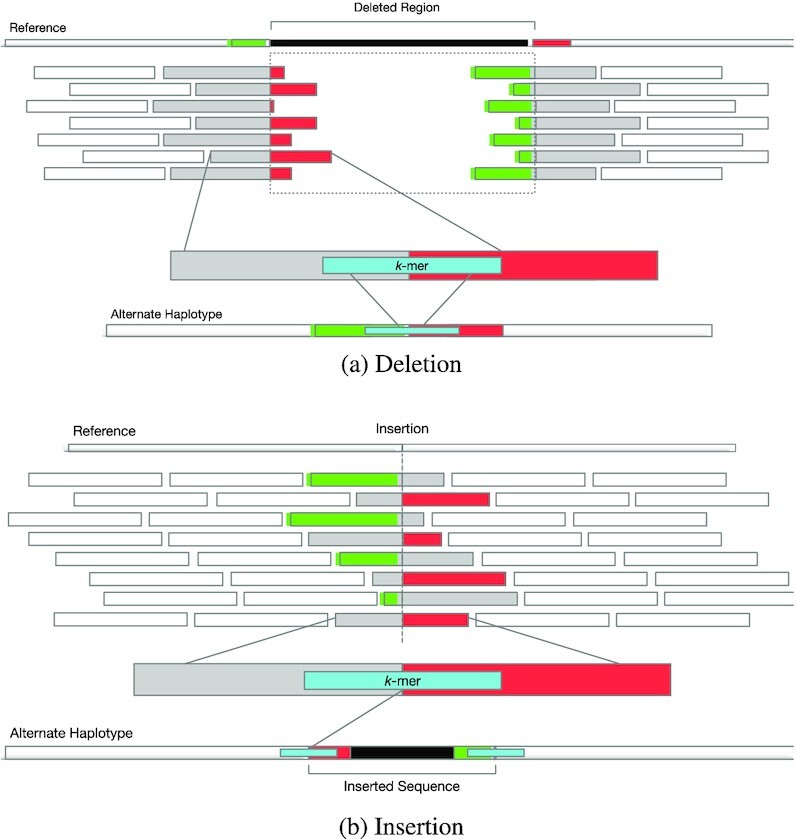
*k*-mer extraction clipped reads for a deletion (**A**) and an insertion (**B**). Red and green segments of the reads are soft-clipped by the aligner and correspond to the similarly colored regions of the alternate and reference haplotypes.

Sequencing reads that cross a SV’s breakpoint are usually soft-clipped when mapped to the reference genome. For each SV, Nebula looks at soft-clipped reads mapping near its breakpoints and selects *k*-mers that overlap the clipped part of the read (Figure 2).

Nebula also uses the reference genome to extract additional *k*-mers from within the region that is affected by a SV (e.g. inside the deleted region for a deletion or from the sequence that would be inserted into the genome for an insertion). We also extract unique *k*-mers that cross the breakpoints from the reference genome.

With the *k*-mers selected, Nebula scans the reference genome to filter any *k*-mer that also occurs in loci not impacted by the input SVs. Finally, the remaining *k*-mers are counted on each of the input sample with known SV genotypes. We use each *k*-mer independently to genotype its corresponding SV and filter those *k*-mers that do not predict the correct genotype. After filtering, the remaining *k*-mers are exported as the output of the *k*-mer extraction phase.

The likelihood of genotype *g* based on *k*-mer *k* with count *c*_*k*_ is calculated using the normal distribution as $\mathcal {L}(g | k) = p(k | g) = p(c_k | \mathcal {N}(\mu _{k,g}, \sigma ^2_{k,g}))$ where $\mu_{g, k}$ and $\sigma ^2_{g, k}$ are derived from the sample-wide mean $\mu$ and variance $\sigma^2$ based on the expected copy number of the *k*-mer *k* for genotype *g*. For example, for an insertion SV, a *k*-mer selected from the inserted sequence is expected to be present on both haploids for a 1/1 genotype with $\mu_{1/1, k} = \mu$ and $\sigma ^2_{1/1, k} = \sigma ^2$ and on only one haploid for a 1/0 genotype with $\mu_{1/0, k} = \mu/2$ and $\sigma ^2_{1/0, k} = \sigma ^2/2$. We calculate the likelihood of all three possible genotypes using the above formulation and choose the one with the maximum likelihood as the genotype prediction.

### Genotyping

Once *k*-mers have been extracted for a set of SVs, the same set of SVs can be genotyped on any new WGS sample(s) without the need to map the reads. The *k*-mers are counted on the sample and genotypes are predicted using an extension of the likelihood model. For a SV supported by multiple *k*-mers, the likelihood of each possible genotype *g* ∈ {0/0, 0/1, 1/1} can be calculated as $\mathcal {L}(g| k_1, k_2, k_3, ... ) = p(k_1, k_2, k_3, ...|g)$ where each *k*_*i*_ represents a different *k*-mer. Note that the counts of *k*-mers corresponding to the same SV might not be independent as the *k*-mers may overlap one another. However, if we assume independence between *k*-mer counts, we can approximate the above likelihood by calculating the probability as the multiplication of probabilities of individual *k*-mers given the genotype (i.e. $\Pi_ip (k_i|g)$). Note that *p*(*k*_*i*_|*g*) is calculated as $p(c_{k_i} | \mathcal {N}(\mu _{g, k_i}, \sigma _{g, k_i}^2))$ where the random variable $c_{k_i}$ is the count of *k*-mer *k*_*i*_ in the sample. Furthermore, the values $\mu _{g, k_i}$ and $\sigma _{g, k_i}^2$ are derived from sample-wide $\mu$ and $\sigma$ according to the genotype *g*. We calculate the likelihood for all three possible genotypes 1/1, 1/0 and 0/0 for each SV and choose the one with the maximum likelihood as our prediction.

### Implementation

Nebula is implemented entirely in C++ and is heavily parallelized using OpenMP (34). To improve speed and reduce memory usage, *k*-mers are hashed into integer values and string comparison operations are implemented in binary arithmetic. This allows Nebula to count millions of *k*-mers in WGS reads at a rate of >500 000 reads per second using a single processor core.

To increase the accuracy of *k*-mer counts, Nebula keeps the immediate left and right *k*-mers surrounding a selected *k*-mer during the extraction phase and checks that at least one of these *k*-mers exists around the *k*-mer in a sequencing read before incrementing the count. This is discussed in more detail in the Supplementary Materials.

Although Nebula is meant to genotype unmapped FASTQ files, *k*-mers can also be counted in SAM, BAM and CRAM files with slight differences in performance between the different formats due to parsing and decoding. Unlike many mapping-based tools that require certain fields in input VCF files, Nebula only requires the SV coordinates (and optionally the inserted sequence for insertions). For the experiments presented in this manuscript, we have also developed a Docker version of Nebula that can be easily deployed to various cloud computing platforms such as Cancer Genomics Cloud (35).

## RESULTS

We utilized both simulations and real data to quantify and evaluate the performance of Nebula using high quality SV predictions from long-read sequencing data on 1KG samples HG00514 (CHS trio, child), HG00733 (PUR trio, child) and NA19240 (YRI trio, child) (36).

### Simulation

An extensive WGS simulation was performed to evaluate Nebula’s performance for accurately genotyping SVs. The simulation consists of two stages: first we mutated a genome with the set of SVs from the 1KG dataset and used it for *k*-mer extraction. Second, we simulated a subset of these SVs on a new sample and used the extracted *k*-mers to genotype the simulated SVs.

During the first step, a diploid GRCh38 genome was mutated with the union of all insertions and deletions reported for samples HG00514 and HG00733 (11551 total SVs) with random genotype assignments of 1/0 or 1/1. Short paired-end sequencing reads were simulated from this diploid simulated genome using wgsim (https://github.com/lh3/wgsim) at 30x coverage and mapped using BWA-mem (37). After running the *k*-mer extraction phase, Nebula found *k*-mers to genotype 11330 (98%) of the simulated SVs.

During the second stage of the simulation, another diploid genome was constructed from GRCh38 and was randomly mutated with the same set of SVs but with all three possible genotypes (0/0, 0/1 and 1/1) allowed. Paired-end short reads were generated from this genome at 30× coverage in FASTQ format and the extracted *k*-mers were used to genotype the sample.

The entire procedure was also repeated at 10× coverage to measure Nebula’s resilience to low coverage. For the 10× simulation, *k*-mers could be extracted for 11304 (97.8%) SVs.

We compared Nebula’s predictions against those of the mapping-based approaches SVTyper (16) and Delly (11), the graph-based approach Paragraph (38) and the *k*-mer-based approach BayesTyper (39). Due to limitation of SVtyper and Delly on genotyping long insertions (40), we have excluded these tools from the comparison for insertions. Note that none of the mentioned methods except BayesTyper can genotype unmapped samples in FASTQ format directly and instead require mapped reads as input.

**Figure 3.**
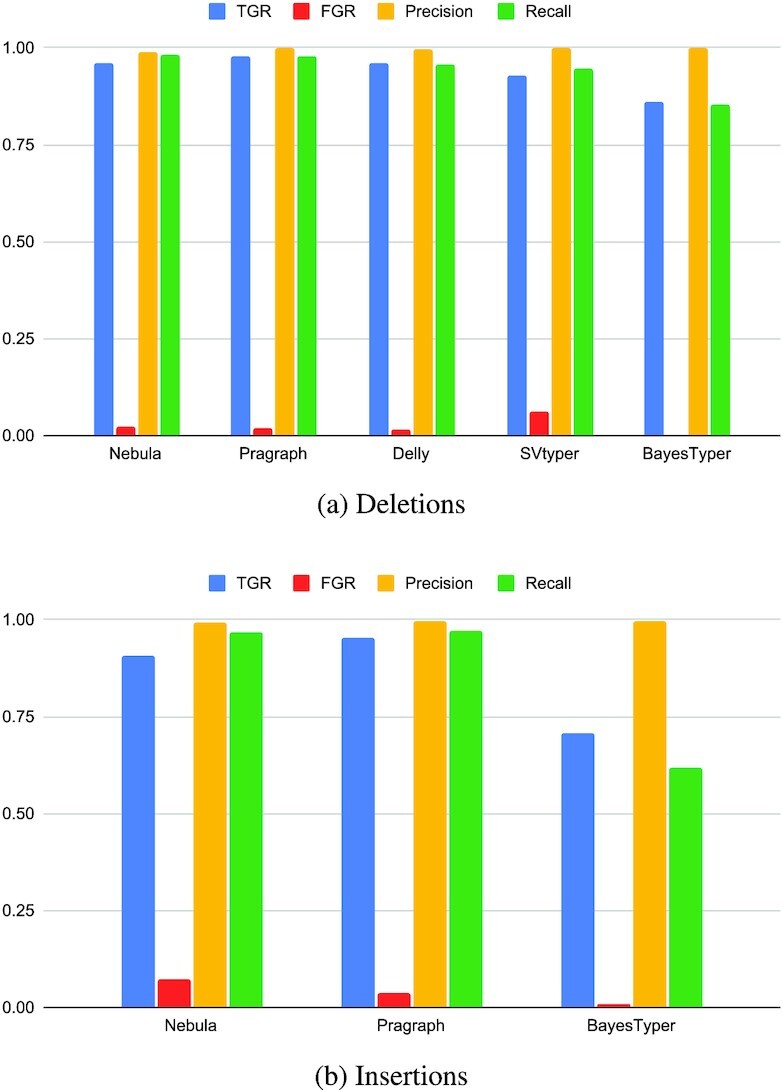
Comparison of different accuracy metrics between Nebula and other methods when genotyping SVs on the 30× simulation.

We calculated four different measures of accuracy for each method: The *true genotyping rate* (TGR) is defined as the number of correct genotype calls for each tool divided by the total number of input events. The *false genotyping rate* (FGR) is similarly defined as the number of false genotype calls made by a tool divided by the total number of calls made by that tool. *Precision* is defined as the number of true positive calls divided by all the positive calls (1/1 and 1/0) made by a tool and finally *recall* is defined as the number of true positive calls produced by a tool divided by the total number of 1/1 or 1/0 SVs present on the sample. The detailed results for each simulation, separated by event type are presented in Figure 3 and Supplementary Figure S1. In both simulations, Nebula has produced comparable results to state-of-the-art genotyping approaches without requiring the mapping of the reads to the reference genome.

### Real data

We also used real WGS data for experimental evaluation of Nebula. We considered the union of all insertions, deletions and inversions reported from non-repeat regions of the HG00514 and HG00733 genomes as the set of input SVs (36). We used these two samples to extract *k*-mers for the SVs and used the *k*-mers to genotype a third sample NA19240 with Nebula. We also used Delly, SVTyper, Paragraph and BayesTyper to genotype the selected set of SVs on NA19240 and validated their predictions against the 1KG callset.

For evaluation, we only considered SVs that could be correctly genotyped on HG00514 and HG00733 using at least one of the four methods (Delly, SVTyper, Paragraph and BayesTyper) in the comparison. For consistency in validating genotypes, we have merged overlapping deletions and insertions (less than 10bp apart) in different samples into a single event. A total of 4810 deletions, 7511 insertions and 81 inversions were considered for our evaluation.

**Figure 4.**
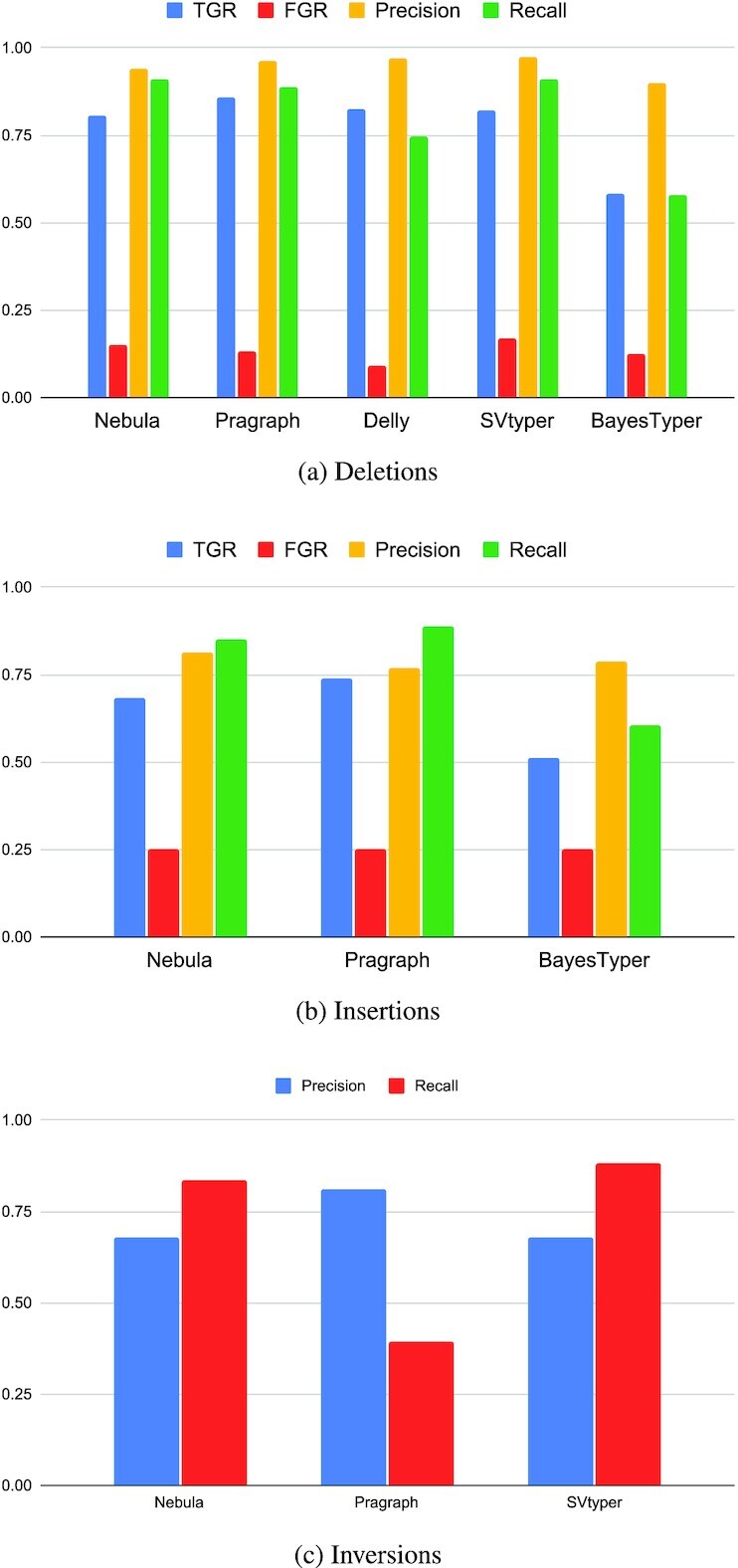
Comparison of different accuracy metrics between Nebula and other methods when genotyping SVs on NA19240.

We use the same metrics introduced earlier for comparing the performance of different methods and we observe that Nebula consistently performs equal to or better than the other state-of-the-art methods (Figure 4). As the input callset does not include genotypes for inversions and only marks them as present or not, we have only reported precision and recall for inversions. We could not genotype the inversions using Delly or BayesTyper and we have thus removed these tools from the comparison for inversions. Note that BayesTyper requires exact SV breakpoints for optimal performance; as a result, its performance for insertions and deletions may have been negatively affected due to inexact breakpoints for some of the SVs in the dataset.

We also compared the performance of different tools for genotyping SVs in repeat regions of the genome. We used each method to genotype SVs reported in HG00514 and HG00733 on the NA19240 sample. Nebula and other methods perform relatively well on SVs involving mobile elements (e.g., SINE or LINE elements) and all methods achieve a precision of over 90% (Supplementary Figure S2). However, on SVs incorporating genomic satellite and tandem repeat regions all tools perform relatively poorly with every tool having a FGR of at least 40% (Supplementary Figure S3). Our results indicate novel methodological developments are required to accurately genotype these types of SVs.

### Time and memory performance

**Figure 5.**
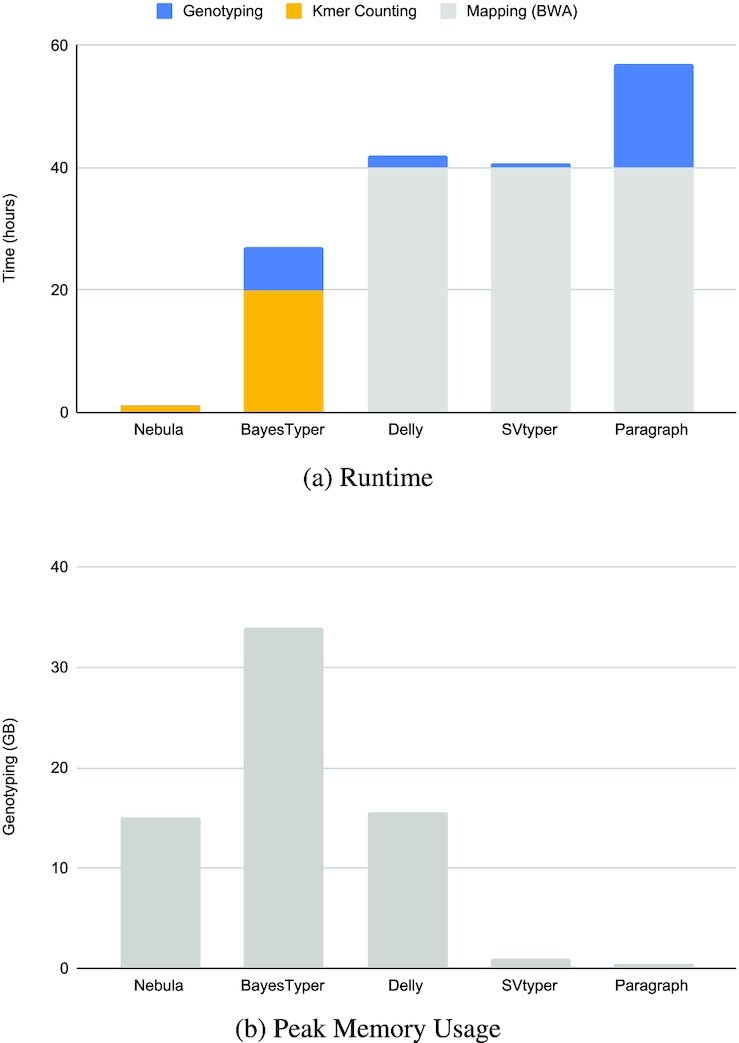
Comparison of single-thread runtime (**A**) and peak memory usage (**B**) of Nebula and other genotyping tools while genotyping 12321 insertion and deletion SVs on unmapped NA19240 reads. Nebula and BayesTyper are *k*-mer-based methods and don’t require read-mappings. Delly and SVtyper mainly parallelize over the number of input samples and don’t benefit from multiple threads when genotyping a single sample. Peak memory usage excludes the memory usage of BWA-mem (peak memory usage of BWA-mem mapping was 16GB).

Nebula’s main advantage is its ability to directly genotype unmapped samples with high efficiency and comparable accuracy to the state-of-the-art mapping-based genotypers. Furthermore, Nebula is not limited to specific types of SVs and can genotype deletions, insertions, inversions, or other types of SVs using a universal algorithm. We measured the runtime and peak memory usage of Nebula and other tools for genotyping NA19240 (Figure 5).

Assuming *k*-mers have already been extracted for a common set of SVs, Nebula can be as much as 40 times faster than mapping-based methods in genotyping newly sequenced samples. This is particularly useful in large studies with hundreds to thousands of samples, where Nebula can be efficiently used to genotype common SVs on the entire cohort an order of magnitude faster than other approaches.

Nebula also has advantages when genotyping mapped samples. For a mapping-based genotyper, the sequencing reads should be mapped against the same reference genome version that the SV coordinates are from; however, once Nebula has extracted *k*-mers for a set of SVs reported against a certain reference genome (e.g. GRCh38), it can genotype them on samples mapped to other reference genome versions (e.g. GRCh37) directly and without the need to remap the samples or lift SV coordinates.

### Simons Genome Diversity Project Data (SGDP)

We used *k*-mers extracted for a total of 14103 insertion and deletion selected from the three 1KG samples HG00514, HG00733 and NA19240 to genotype the entire set of 279 samples from the Simons Genome Diversity Project (41) stored in BAM format on the cloud computation platform Cancer Genomics Cloud (CGC) (35).

**Figure 6.**
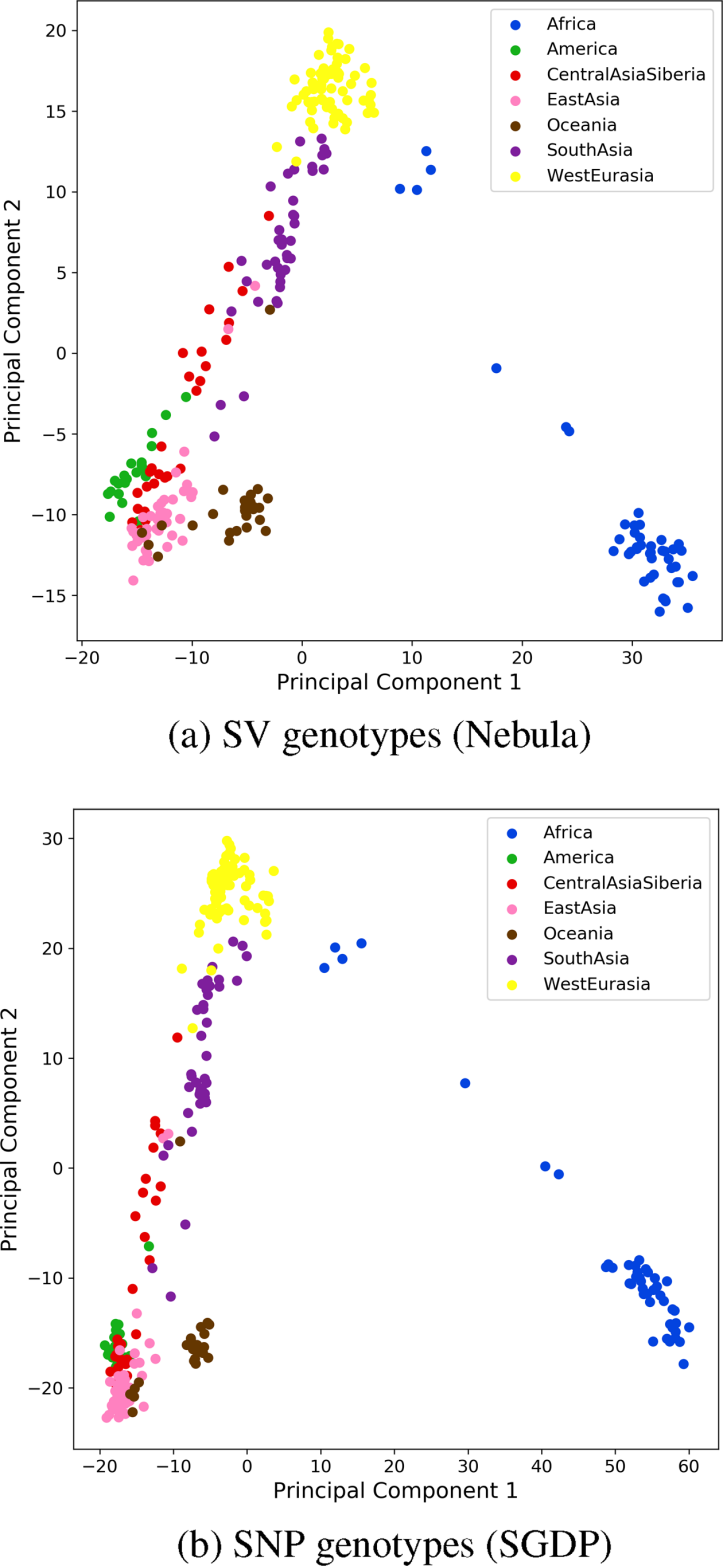
Population clustering of SGDP samples.

On average, we see about $19\%$ homozygous and $24\%$ heterozygous genotype predictions among all samples. We expect the genotypes to cluster samples based on geographical origin. For this, we preformed a principal component analysis (PCA) on the SV genotypes and plotted the two most significant components (Figure 6). The PCA clearly separates populations of different continents with a greater level of separation between Africa and the rest (Figure 6A). We repeated the PCA analysis using one million randomly selected SNP calls from the Simons Genome Diversity Project (41) and plotted the two most significant components (Figure 6B). The plot from Nebula’s genotypes captures the same structure as SGDP’s SNP calls, showing the accuracy of our method for population studies.

The 1KG SVs are based on GRCh38 coordinates, however the SGDP samples are mapped against GRCh37. With Nebula’s modest resource requirements and independence from mapping, each sample was genotyped accurately in under an hour and at a cost of $0.30 per sample without the need to remap the reads to GRCh38.

## DISCUSSION

We have presented here, Nebula, a novel approach for ultra-efficient and accurate genotyping of any type of SV without the need to map the reads to the reference genome. We have demonstrated that *k*-mers can act as a lightweight and simple alternative for expensive mapping-based methods to genotype polymorphic SVs. Several tools have already achieved similar conclusions for other types of variants such as SNVs (26,27) and indels (21,28). Furthermore, our proposed approach can easily be modified to genotype other types of variants (i.e. SNVs and indels). Thus, we believe that utilizing a combination of these mapping-free methods can provide a framework for accurate and efficient genotyping of all types of variation using *k*-mer counts. This would significantly reduce the computational resources needed to analyze new WGS samples and will speed-up large scale studies.

Note that Nebula does not require exact SV breakpoints for genotyping SVs and can work with approximate breakpoints. This is an advantage over approaches that require exact breakpoints or assembled haplotypes to guide *k*-mers selection and accurate variant genotyping. Nebula only counts the *k*-mers directly associated with the SVs, significantly reducing the runtime and memory usage compared to other *k*-mer based approaches.

Furthermore, genotype imputation algorithms (42) can be incorporated into Nebula’s pipeline to improve the method’s accuracy and ability to genotype variants that are difficult to genotype using solely *k*-mers, e.g. SVs with breakpoints in repeat regions of the genome.

Finally, extending Nebula to utilize *k*-mers that are shared between different SVs may help us improve our performance when genotyping SVs in repeat regions of the genome (e.g. tandem repeats).

## DATA AVAILABILITY

The code and data used in these experiments are available at https://github.com/Parsoa/Nebula.

## Supplementary Material

gkab025_Supplemental_File
