# Supplementary material for: Nebula: ultra-efficient mapping-free structural variant genotyper
Source: Nucleic Acids Res. 2021 Jan 27;49(8):e47. doi: 10.1093/nar/gkab025 (PMC8096284; doi:10.1093/nar/gkab025)
Supplement: gkab025_Supplemental_File [file gkab025_supplemental_file.pdf]

Supplementary Material

k-mer counting implementation details

Nebula only works with unique k-mers, e.g k-mers that are only seen in one loci in the genome and are as a result associated with a single SV. However to increase the number of available k-mers and improve the accuracy of counting, we use a broader definition of a unique k-mer in our implementation which also takes into account the context surrounding the k-mer. When extracting k-mers, Nebula stores the immediate left and right 32bp sequences surrounding a k-mer as "masks". A k-mer is considered unique if it is associated with only one SV and the combination of the k-mer and its masks, 96bp in total, is only seen in one loci in the genome. In this sense, a k-mer could be present in multiple loci outside of the SV in the genome, but as long as the SV locus has unique masks that are not seen in any other non-SV loci, the k-mer is still considered unique. k-mers whose SV locus cannot be uniquely identified using the masks are discarded.

When counting k-mers in the reads, Nebula checks for exact matches for the k-mers, however once a k-mer is found in a read, only approximate matches are required for the masks, i.e only 28bp of each mask needs to match. With short sequencing reads being typically shorter than 120bp, it's unlikely to see a k-mer and both masks in a read, instead the presence of a single masks is enough, as long as the mask uniquely identifies the SV locus among all all loci of the k-mer.

As it is possible for a unique k-mer to appear as a result of SNPs or sequencing errors, the use of masks prevents counting of such instances, potentially reducing the possibility of overestimating k-mer counts. However as k-mers need to exactly match to be counted, Nebula may miss instances of k-mers affected by errors or SNPs and hence it is possible for a k-mer's count to be underestimated. This is a trade-off between speed and accuracy.

Comparison results for the 10x simulation

Figure 1 shows the comparison of different accuracy metrics between Nebula and other tools when genotyping the 10x simulation. The lower coverage in the 10x sample results in a higher FGR for Nebula compared to the 30x simulation, as the separation of 1/1 and 1/0 genotypes based on k-mer counts has a smaller margin. However Nebula's accuracy metrics remain on-par with other methods, showing that the method is robust to low sequencing depth.

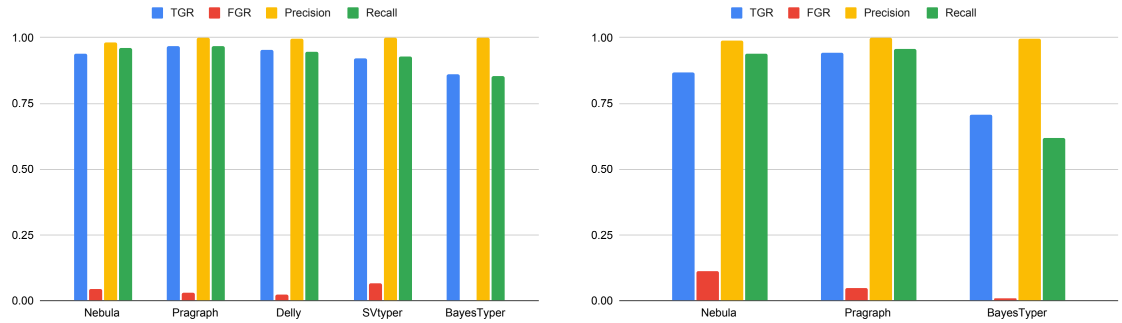

(a) Deletions

(b) Insertions

Supplementary Figure 1: Different performance metrics calculated for all tested tools for deletions (a) and insertions (b) on the 10x simulation.

Comparison results for mobile element polymorphisms on NA19240

Figure 2 below shows the performance of Nebula and other considered approaches when genotyping SVs categorized as transposable elements (`SVCLASS=ALU,L1,LTR,HERV` in the VCF files) on NA19240. Mobile

element polymorphisms can manifest both as deletions and insertions. The SVs considered in this figure are a subset of those shown in Figure 4. Delly and SVTyper are not included in the comparison due to their limitation in genotyping insertions.

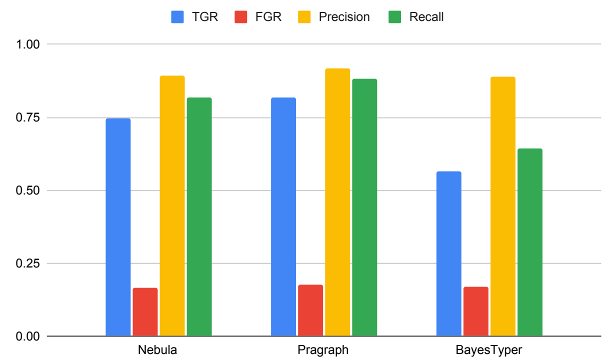

Supplementary Figure 2: Comparison of different accuracy metrics between Nebula and other genotyping tools for genotyping MEIs on NA19240. Includes both repeat and non-repeat regions.

Comparison results for SV calls in tandem repeat and satellite regions on NA19240

We repeated the experiment on NA19240 using the SVs reported on tandem repeat and satellite regions of the HG00514 and HG00733 genomes, i.e those with `IS_TRF=TRUE` in the VCF file from 1KG. The comparison can be seen in Figure 4. All tools perform relatively poorly; no method achieves a TGR above 60% on deletions and all methods have a less than 50% TGR on insertions. Still Nebula achieves the highest TGR on insertions and the lowest FGR on deletions.

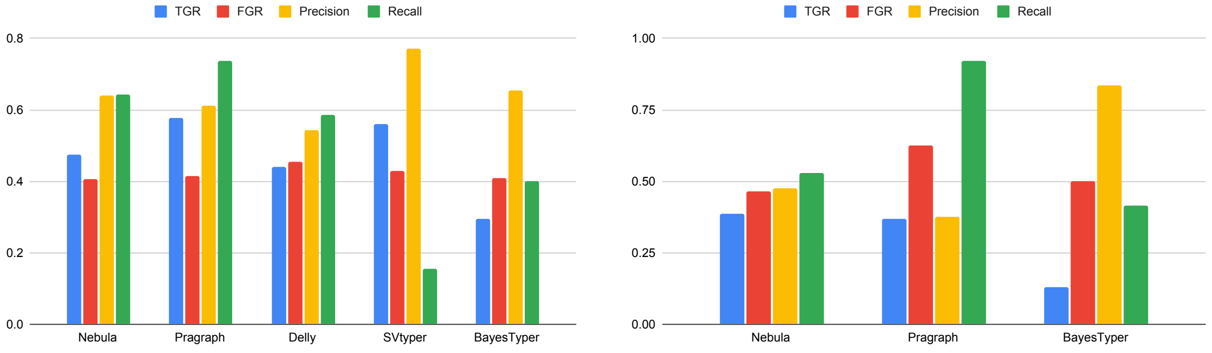

(a) Deletions (b) Insertions

Supplementary Figure 3: Comparison of different accuracy metrics between Nebula and other genotyping tools for repeat events on NA19240.
